# Regional Dissemination of a Trimethoprim-Resistance Gene Cassette via a Successful Transposable Element

**DOI:** 10.1371/journal.pone.0038142

**Published:** 2012-05-30

**Authors:** Amy S. Labar, Jennifer S. Millman, Ellen Ruebush, Japheth A. Opintan, Rima A. Bishar, A. Oladipo Aboderin, Mercy J. Newman, Adebayo Lamikanra, Iruka N. Okeke

**Affiliations:** 1 Department of Biology, Haverford College, Haverford, Pennsylvania, United States of America; 2 Department of Microbiology, University of Ghana Medical School, Accra, Ghana; 3 Department of Medical Microbiology and Parasitology, Obafemi Awolowo University, Ile-Ife, Nigeria; 4 Department of Pharmaceutics, Obafemi Awolowo University, Ile-Ife, Nigeria; U. S. Salinity Lab, United States of America

## Abstract

**Background:**

Antimicrobial resistance is a growing international problem. We observed a 50% increase in the prevalence of trimethoprim resistance among fecal *Escherichia coli* from healthy Nigerian students between 1998 and 2005, a trend to increase that continued in 2009.

**Methods and Findings:**

A PCR-based screen revealed that 131 (43.1%) of isolates obtained in Nigeria in 2005 and 2009 carried integron-borne *dfrA* cassettes. In the case of 67 (51.1%) of these isolates, the cassette was a class 1-integron-borne *dfrA7* gene, which has been reported at high prevalence from *E. coli* isolates from other parts of Africa. Complete sequencing of a 27 Kb *dfrA7*-bearing plasmid from one isolate located the *dfrA7* gene within a Tn*21*-type transposon. The transposon also contained an IS*26*-derived *bla/sul/str* element, encoding resistance to β-lactams, sulphonamides and streptomycin, and mercury resistance genes. Although the plasmid backbone was only found in 12 (5.8%) of trimethoprim-resistant isolates, *dfrA7* and other transposon-borne genes were detected in 14 (16.3%) and 32 (26.3%) of trimethoprim resistant isolates collected in Nigeria in 2005 and 2009, respectively. Additionally, 37 (19.3%) of trimethoprim-resistant *E. coli* isolates collected between 2006 and 2008 from Ghana were positive for the *dfrA7* and a transposon marker, but only 4 (2.1%) harbored the plasmid backbone.

**Conclusions:**

Our data point to transposition as a principal mechanism for disseminating *dfrA7* among *E. coli* from Nigeria and Ghana. On-going intensive use of the affordable broad-spectrum antibacterials is likely to promote selective success of a highly prevalent transposable element in West Africa.

## Introduction

The broad-spectrum antibacterial trimethoprim is heavily used worldwide, most commonly in combination with sulphonamides. Following the evolution of resistance trimethoprim has, in many cases, been replaced by newer antimicrobials. But because of its low cost, broad spectrum, excellent safety profile, high stability and oral bioavailability, trimethoprim-sulphamethoxazole is still used intensively in many African countries [1], [2]. In the last two decades trimethoprim-sulphamethoxazole has been recommended as a prophylactic in HIV/AIDS patients on top of extensive curative use. This medicament has prevented potentially life-threatening opportunistic infections, is a useful prelude to antiretroviral therapy for many patients and averts loss of life that would otherwise accompany the slow roll-out of antiretrovirals [3], [4].

**Figure 1:**
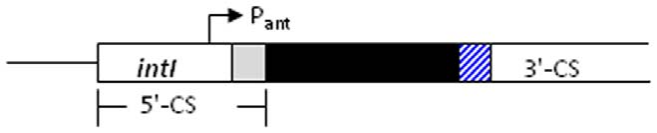
Structure of generic integron. Each integron is comprised of 5′ and 3′ conserved ends separated by a variable region (black), which contains zero to several cassettes. The figure illustrates an integron bearing a single cassette. Cassette incorporation occurs by recombination between an *attC* attachment site at the 3′ end of the the gene cassette (hatched) at the integron’s attachment site (*attI*), located at the 5′ conserved end. This reaction is catalyzed by a site-specific recombinase encoded by the 5′ *intI* gene. Class 1 and Class 2 integrons, which are the sub-categories most commonly associated with drug resistance contain a promoter at the 5′ conserved end, from which cassette genes are transcribed.

Trimethoprim inhibits the enzyme dihydrofolate reductase (Dfr). *E. coli* is most commonly becomes resistant by acquiring one of over 30 known *dfr* genes encoding resistant variants, most of which belong to the *dfrA* category [5]. Many *dfr* gene cassettes, including about half of the known *dfrA* alleles, lie within gene capture and expression systems known as integrons [6]. Integrons contain an integrase gene, the product of which catalyzes the integration of circular cassettes at an adjacent integration site, *attI*. Each antimicrobial resistance cassette consists of a promoterless gene and an attachment site, *attC*, which recombines with *attI* upon integration (Figure 1). A given integron can have from zero to several cassettes and cassette transcription is facilitated by a promoter at the 5′ end of the integration site. Four classes of integrons have been well-characterized, although others do exist in nature [7]. Class 1 and class 2 integrons have been implicated in resistance gene dispersal, particularly in *E. coli* and other enteric bacteria.

For three decades, the class 2 integron-bearing transposon Tn*7* has been implicated in global dissemination of the *dfrA1* gene across a wide range of bacterial species [8], [9], [10], [11], [12], [13], [14], [15]. There have since been many studies cataloguing the relative distribution of different *dfr* cassettes across the globe but, in spite of increasing interest in the forces underlying evolution to trimethoprim resistance [16], [17], [18], very little is known about the context in which more than a handful of the most promiscuous alleles are spread. Trimethoprim resistance rates among commensal and pathogenic enteric bacteria reported in the literature from Africa are very high, typically approaching or exceeding 60% [19], [20], [21], [22], [23], [24]. In 2005, we observed an increase in trimethoprim resistance among fecal *E. coli* from healthy adults in Nigeria compared to similar strain collections procured in 1986–1998. We sought to determine the contributions of integrons to multiple- and trimethoprim resistance. Trimethoprim resistance was most commonly attributed to a class 1 integron-borne *dfrA7* cassette. We locate this promiscuous cassette to a transposable element and demonstrate that *dfrA7* cassettes on mobile elements may be regionally disseminated.

## Materials and Methods

### Strains

Permission to conduct this study was provided by the Institutional Review Board of Obafemi Awolowo University, Ile-Ife, Nigeria. The study was also approved by the Institutional Review Board of the University of Ghana Medical School. All volunteers gave informed consent. *E. coli* isolates were recovered from stool specimens collected from apparently healthy undergraduates at the Obafemi Awolowo University and from apparently healthy adults presenting for physical check-ups at the Korle Bu Hospital Teaching Hospital as described previously [20], [25]. We collected and processed specimens in 2005 and 2009 using identical protocols to those used in 1986–1998 in Nigeria and 2006–2008 in Ghana [20], [25]. Lactose-fermenting colonies isolated on MacConkey agar were confirmed as *E. coli* by biochemical testing. (Lactose-negative *E. coli* were excluded). Colonies from the same specimen with identical biochemical and susceptibility profiles were treated as identical isolates. A total of 128 isolates from Nigeria in 2005 and 176 in 2009 were included along with 130 isolates from Ghana in 2006, 73 isolates from Ghana in 2007, and 88 isolates from Ghana in 2008.

### Susceptibility Testing

The Clinical and Laboratory Standards Institute (CLSI, formerly NCCLS) disc diffusion method was used to determine susceptibility of the isolates to eight antibacterials [26]. Discs used contained ampicillin (10 µg), streptomycin (10 µg), trimethoprim (5 µg), tetracycline (30 µg), nalidixic acid (30 µg), chloramphenicol (30 µg), ciprofloxacin (5 µg) and sulphonamide (300 µg) (Oxoid/Remel). *E. coli* ATCC 35218 was used as control strain. Inhibition zone diameters were interpreted in accordance with CLSI guidelines using WHONET software version 5.3 [27].

### General Molecular Biology Procedures

Genomic DNA was extracted using the Promega Wizard kit. DNA amplification was performed using Platinum PCR Supermix (Invitrogen) and 1 µM oligonucleotide primer in each reaction. Oligonucleotide primer sequences are listed in Table S1. Sensitivity and specificity of each primer pair for its target sequence was tested *in silico*, using Genbank and BLAST, and experimentally using strains of known genotype. All amplifications began with a two-minute hot start at 94°C followed by 30 cycles of denaturing at 94°C for 30 s, annealing at 5°C below primer annealing temperature for 30 s, and extending at 72°C for one minute per kilobase of DNA. When the target PCR product was over 3 Kb, we used *Pfx* polymerase in accordance with manufacturer’s instructions, with annealing at 5°C below the temperature used for *Taq* PCRs. Where necessary, for sequencing, PCR amplicons were TA cloned into the pGEMT vector (Promega) according to manufacturer’s directions and plasmids were transformed into chemically competent *E. coli* K-12 TOP10 cells. Large, naturally occurring plasmids were electroporated into DH5αE electrocompetent cells (Invitrogen) using a Biorad micropulser according to manufacturers’ instructions, and extracted by a modified boiling protocol as described previously [28], [29]. All other molecular biology operations were performed using standard procedures [30].

### Integron Cassette Amplification and Identification

The variable, cassette-containing portions of class 1 and class 2 integrons were amplified using conserved-end primers and protocols described by Levesque et al [31] and White et al [32] respectively. Restriction fragment polymorphism analyses of all amplicons were performed in separate RFLP reactions using *Alu*I and *Mbo*I. At least three representatives of unique profiles were cloned and sequenced.

### Plasmid Replicon Typing

Three multiplex panels comprised of 18 primer pairs were used to identify plasmid replicons by PCR as described by Johnson et al [33]. Strains carrying well-characterized plasmids pMAR-7, pB171, pHCM1 and pED204 were used as controls [34], [35], [36], [37].

### *fliC* Genotyping and Multilocus Sequence Typing

*fliC* PCR-restriction fragment length polymorphism (RFLP) typing was performed as described by Fields et al [38]. The *E. coli fliC* gene was amplified using the primers F-FLIC1 and R-FLIC2 (Table S1). Amplicons were digested with *Rsa*I and restriction profiles were compared after electrophoresis on 2.5% agarose gels. Multilocus sequence typing was performed as described by Wirth et al [39]. PCR primers listed in Table S1 were used to amplify gene fragments from the *adk, fumC, gyrB, icd, mdh, purA* and *recA* and amplified DNA products were sequenced from both ends. Allele assignments were made at the publicly accessible *E. coli* MLST database at http://www.mlst.net/.

### Shot-gun Sequencing and Sequence Analysis

Whole-replicon shotgun library preparation, sequencing and assembly of a large plasmid were performed by SeqWright DNA Technology Services (Houston, TX). Sequence analyses and annotation were performed in Artemis [40]. Open reading frames were initially defined by Glimmer. Annotations were made where BLAST e-values equaled or approached zero and there was 98% or greater identity at the DNA and amino acid levels. Direct and inverted repeats were identified by dot-plot analysis of pairwise FASTA alignments made using the BLAST suite. Open reading frame and feature plots were prepared using Artemis and DNAPlotter [41].

## Results and Discussion

### Increase in Trimethoprim Resistance Rates in South-western Nigeria from 2005 is Associated with a Predominant, Class 1-integron-borne *dfrA7* Cassette

**Figure 2:**
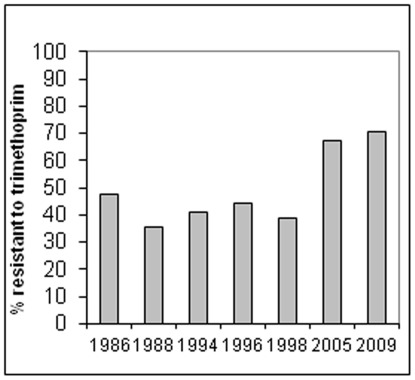
Trimethoprim resistance trends among *E. coli* isolates from healthy adults in Ile-Ife Nigeria 1988–2009.

Between 1986 and 1998, we followed resistance trends for eight antimicrobials among commensal bacteria belonging to the species *E. coli* from apparently healthy Nigerian students [25]. We observed a temporal increase in resistance for most heavily used agents with the notable exception of trimethoprim, a drug commonly administered as a fixed combination with sulphamethoxazole. During this period, resistance to trimethoprim, remained at a stable high of 35–45% [25]. In this study, deliberately designed to replicate the 1986 to 1998 protocols, stool specimens were collected from 83 and 101 consenting adults in 2005 and 2009 respectively [1]. Susceptibility testing of the 304 *E. coli* isolates obtained revealed that resistance to most agents commonly used in Nigeria was high (Table S2). As shown in Figure 2, trimethoprim resistance has become much more prevalent than in previous decades, showing an 80% increase in 2005 on 1998 data, with the number and percentage of trimethoprim-resistant strains increasing again, from 86 (67.2%) to 122 (70.8%), between 2005 and 2009.

We used primers that anneal to the conserved ends of class 1 and class 2 integrons [31], [32] to amplify the interim cassette regions of all 128 *E. coli* isolates collected in Ile-Ife, Nigeria in 2005 and 176 isolates from 2009. These primers identify strains carrying complete and intact integrons but exclude those that may be truncated at the 3′ end or otherwise modified [42]. We identified specific cassettes by sequencing and restriction fragment length polymorphism analyses. Based on data from similar studies elsewhere in the world, we hypothesized that *dfrA* genes would be detected and that *dfrA1*, the most commonly reported allele worldwide [5], [10], [16], [18], [43], [44] would feature predominantly. We did indeed identify only *dfrA* alleles, including several strains harboring *dfrA1*. There were only two varieties of class 2 integron cassettes, both carrying *dfrA1* cassettes. The more common variant was *dfrA1-sat-aadA,* known to be globally disseminated via transposon Tn*7* [16], [45]. However, in contrast to recent studies performed outside Africa [46], [47], *dfrA1* was not the most common allele in class 1 integrons. There was low diversity overall and 67(56.8%) of isolates with a class 1 integron, carried a single *dfrA7* cassette identical to that in Tn*5086*, originally isolated from an *E. coli* strain in Colombo, Sri Lanka [48].

### Class 1 Integron-borne *dfrA7* Cassettes Occur Widely Among *E. coli* from Sub-Saharan Africa

We examined the literature for studies that sought class 1 integron cassettes from intestinal *E. coli*. *dfrA7* alleles in the class 1 integron single-cassette conformation have been reported previously from *E. coli* isolates, but not commonly. Of the over 100 studies that characterized integron cassette contents, most found *dfrA7* relatively uncommon, if geographically widespread. The cassette was identified at low levels in community-acquired *E. coli* in the following countries: Finland (0–2%) [45], [49], Korea (4%) [50], Bolivia (2.6%) [47] Spain (3.6%) [46], Taiwan (3.8%) [51], Greece (0.9%) [52], the USA (4.4%) [17], Lebanon (2%) [53], Australia (1/20) [54] as well as Sweden and Sri Lanka (<7%) [16], [48]. *dfrA7* is also more commonly reported from *Salmonella* [55], [56]). By contrast, the few, generally small studies from Africa, reported *dfrA7* from *E. coli* in much larger proportions: 18.7% in Nigeria [57], 33.3% in Senegal [58], 38% in South Africa [59], and more recently, 49% in the Central African Republic [60]and 40% in Tunisia [61]. Descriptive genetic studies of *E. coli* isolates from Nigeria have invariably found *dfrA7* [62], [63]. In other recent studies across Africa where the specific *dfr* alleles have been uncharacterized, rates of resistance to trimethoprim are high [22], [64]. These data suggest that *dfrA7* may be disseminated sub-regionally.

To test the hypothesis that class 1 integrons bearing *dfrA7* cassettes were also highly prevalent among *E. coli* outside our Ile-Ife, Nigeria study area, we screened 291 isolates collected in Accra, Ghana between 2006 and 2008 [20]. As shown in Table 1, a few cassette combinations were identified repeatedly at one geographic location but not at the other. Future studies could reveal that these combinations represent locally disseminated elements or clones. They included *dfrA5* and *dfrA15* from Nigeria and *dfr2d* in Ghana. However, eight cassette arrays were seen at both sites. In addition to *dfrA1*, these include *dfrA15-aadA1* (reported from *Vibrio spp* in Africa [65], [66]) as well as, *dfrA17-aadA5*, which is associated with uropathogenic clonal group A strains known to be present in Nigeria [24], [67]. However, all of other cassette combinations detected at both sites, combined, were less common than *dfrA7,* which was identified in 54 (52.4%) class 1 integron-bearing isolates.

**Table 1:** Integron cassettes in *E. coli* from Nigeria and Ghana between 2005 and 2009.

	Nigeria 2005 (n = 128)	Nigeria 2009(n = 176)	Ghana 2006 (n = 130)	Ghana 2007 (n = 73)	Ghana 2008 (n = 88)
**Integrons in ** ***E. coli*** ** isolates (%)**					
Single class 1 integron only	37 (28.9)	70 (39.8)	35 (26.9)	23 (31.5)	45 (51.1)
Multiple class 1 integrons	–	4 (2.3)	–	1 (1.3)	–
Class 2 integron only	8 (6.3)	14 (8.1)	6 (6.6)	1 (1.3)	2 (22.7)
Class 2 integron and single class 1 integron	1 (0.8)	13 (7.6)	5 (3.8)	2 (2.7)	9 (10.2)
Class 2 integron and multiple class 1 integron	-	1 (0.6)	–	–	–
**Gene Cassettes in Class 1 Integrons in isolates bearing one integron (%)**	(n = 37)	(n = 74)	(n = 35)	(n = 23)	(n = 45)
*dfrA7*	22 (59.5%)	45 (60.8%)	19 (54.3%)	11 (4.8%)	24 (53.3%)
*dfrA1 aadA1*	4 (10.8%)	11 (14.9%)	3 (8.6%)	5 (21.7%)	12 (26.7%)
*dfrA5*	3 (8.1%)	6 (8.1%)	–	–	–
*dfrA15 aadA1*	3 (8.1%)	–	9 (25.7%)	1 (4.3%)	–
*dfrA17 aadA5*	2 (5.4%)	2 (2.7%)	3 (8.6%)	2 (8.7%)	3 (6.7%)
*aadA5*	1 (2.7%)	–	–	–	–
*dfrA1*	1 (2.7%)	–	–	–	–
*dfrA2d*	–	–	1 (2.9%)	4 (17.4%)	5 (11.1%)
*aadA1*	–	1 (1.4%)	2 (5.7%)	3 (13%)	–
*blaOXA-1 aadA1*	–	3 (4.1%)	1 (2.9%)	–	4 (8.9%)
*dfrA17 aadA4*	–	–	–	–	2 (4.4%)
*dfrA15*		5 (6.8%)			
*aadA2*		1 (1.4%)			
*dfrA12 orfF aadA2*		1 (1.4%)			
**Gene Cassettes in Class 2 Integrons (all)**	(n = 9)	(n = 27)	(n = 11)	(n = 3)	(n = 11)
*dfrA1, sat1*	1 (11.1%)	4 (14.8%)	1 (9.1%)	-	3 (27.2%)
*dfrA1, sat1, aadA1*	8 (88.9%)	23 (85.2%)	10 (82.6%)	3 (100%)	8 (72.7%)

### Independent Isolates from Nigeria and Ghana Bearing *dfrA7* Cassettes are not Clonal

Clonal expansion has accounted for dissemination of some *dfrA* alleles elsewhere on the globe and we hypothesized that it could account for *dfrA7* spread in Nigeria and Ghana [24], [68], [69], [70]. When we examined the genetic background of 12 strains by multi-locus sequence typing (MLST) by the scheme of Wirth et al [39], we identified nine different sequence types (Table S3). Similarly, flagellin typing [38] of 35 other *dfrA7*-positive isolates identified at least 15 different flagellin types among 23 typeable strains (Figure S1), confirming that the genomic background of isolates bearing the *dfrA7* gene is diverse.

### Isolate 05/01a from Nigeria Bears *dfrA7* on a Plasmid Encoding Resistance to Multiple Antimicrobials

We next sought to determine whether the *dfrA7* cassette could be transferred to a nalidixic acid-resistant derivative of *E. coli* K-12 strain C600 by conjugation. We employed seven *dfrA7*-bearing strains as conjugative donors, five from Nigeria and two from Ghana. All were resistant to ampicillin, streptomycin, sulphonamides, tetracycline and trimethoprim, five strains were additionally resistant to chloramphenicol and five showed low-level nalidixic acid resistance. All the isolates tested were ciprofloxacin-sensitive. As shown in Table 2, the *dfrA7* gene could be conjugated from only three of these isolates. Therefore, although we are able to conclude that the *dfrA7* cassette can be conjugated *in vitro* in some instances, it is present in a non-conjugable form in other strains.

**Table 2:** Results of conjugation experiments using *dfrA7*-positive isolates as donors.

Strain	Source (year)	Cassette(s)	Donor Resistance Profile	Trans-conjugant Resistance Profile
*dfrA7*-positive strains				
05/01a	Nigeria (2005)	*dfrA7*	A (N)SLTR	None*
05/9c	Nigeria (2005)	*dfrA7*	AC (N)SLTR	None*
05/23a	Nigeria (2005)	*dfrA7*	AC (N)SLTR	AC NSLTR
05/25a	Nigeria (2005)	*dfrA7*	AC (N)SLTR	None*
05/33a	Nigeria (2005)	*dfrA7*	AC (N)SLTR	AC NSLTR
046	Ghana (2006)	*dfrA7*	A SLTR	A N LTR
116	Ghana (2006)	*dfrA7*	AC SLTR	AC NSLTR
Strains bearing cassettes other than *dfrA7*				
115	Ghana (2006)	*aadA1 dfrA15*	AC L R	AC N LTR
05/27a	Nigeria (2005)	*dfrA17 aadA5*	AC SLTR	AC NLTR
Control strains				
R100.1	[92]	*aadA1*	C SLT	C SLT
SM10	[93]	ND	K	None*

* Indicates that no transconjugants were isolated;

Antibiotic code key: A, ampicillin; C, chloramphenicol; N, nalidixic acid (high level); (N), Nalidixic acid (lower level); S, streptomycin; L, sulfonamides; T, tetracycline; R, trimethoprim; K, Kanamycin.

**Figure 3:**
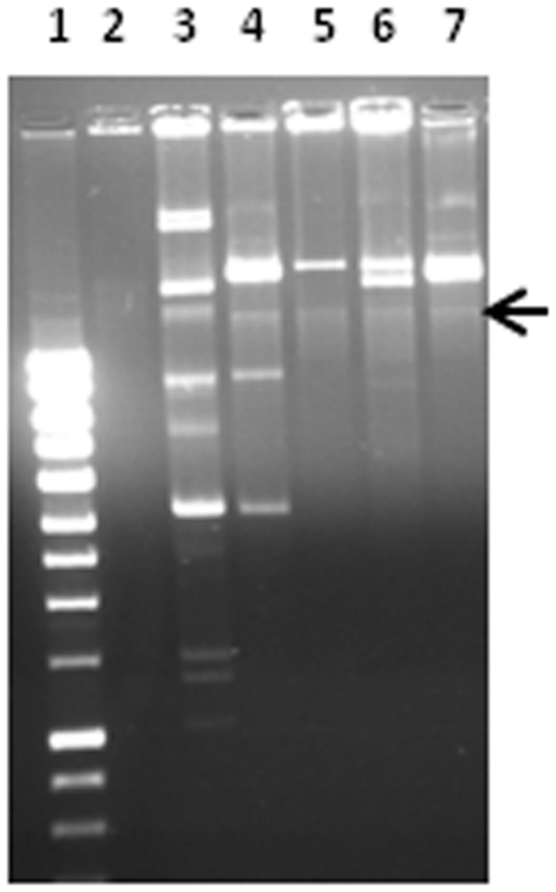
Plasmid profiles of *E. coli* strain 05-01a and its plasmid transformants. Lane 2: Plasmid-free DH5α; Lane 3: 05-01a, *dfrA7*-positive 2005 isolate from Nigeria; Lanes 4–7: four independent transformants produced by electroporating DH5α with a plasmid preparation from 05-01a and selecting on plates containing 50 µg/ml trimethoprim. The arrow indicates the position of chromosomal DNA, as inferred from the DH5α profile. Lane 1: Hyperladder 1 marker.

As shown in Figure 2, *dfrA7-*positive strain 05/01a from Nigeria harbors at least four plasmids. This strain is unable to conjugate *dfrA7 in vitro* (Table 2), but could serve as a transformative donor of the gene (Table 3). Strain 05/01a is susceptible to ciprofloxacin and chloramphenicol but resistant to the other six tested antimicrobials, including low-level resistance to nalidixic acid. Electroporation of a Kado and Liu [28] plasmid extract into DH5α-E cells (Invitrogen) yielded transformants on trimethoprim plates that are co-resistant to ampicilin, streptomycin and sulphonamides but not to nalidixic acid or tetracycline (Table 3). While having different plasmid profiles, each of these transformants carried a single, large, low-copy number plasmid on which the *dfrA7* gene is located (Figure 3). As shown in Table 3, the plasmid in question could not be incompatibility-typed with the primers of Carattoli et al [71], which represent the most common plasmid incompatibility groups.

**Table 3:** Plasmid replicons detected in select *dfrA7*-bearing strains isolated from Nigeria in 2005.

Strain	Resistance profile	Integron-borne trimethoprim resistance gene	Plasmid replicons detected by PCR
05/01a	A (N)SLTR	*dfrA7*	T, Y
05/09c	AC (N)SLTR	*dfrA7*	T, Y
05/23a	AC (N)SLTR	*dfrA7*	FIC, T, Y
05/30a	AC (N)SLTR	*dfrA7*	FIB, T, Y
05/31a	AC (N)SLTR	*dfrA7*	–
05/32c	AC (N)SLTR	*dfrA7*	B/O, FIB, Y
05/33a	AC (N)SLTR	*dfrA7*	B/O, T
DH5αE (pASL01a)	A SLR	*dfrA7*	–
DH5αE	–	None	–

Antibiotic code key: A, ampicillin; C, chloramphenicol; (N), Nalidixic acid (low level); S, streptomycin; L, sulfonamides; T, tetracycline; R, trimethoprim.

**Figure 4:**
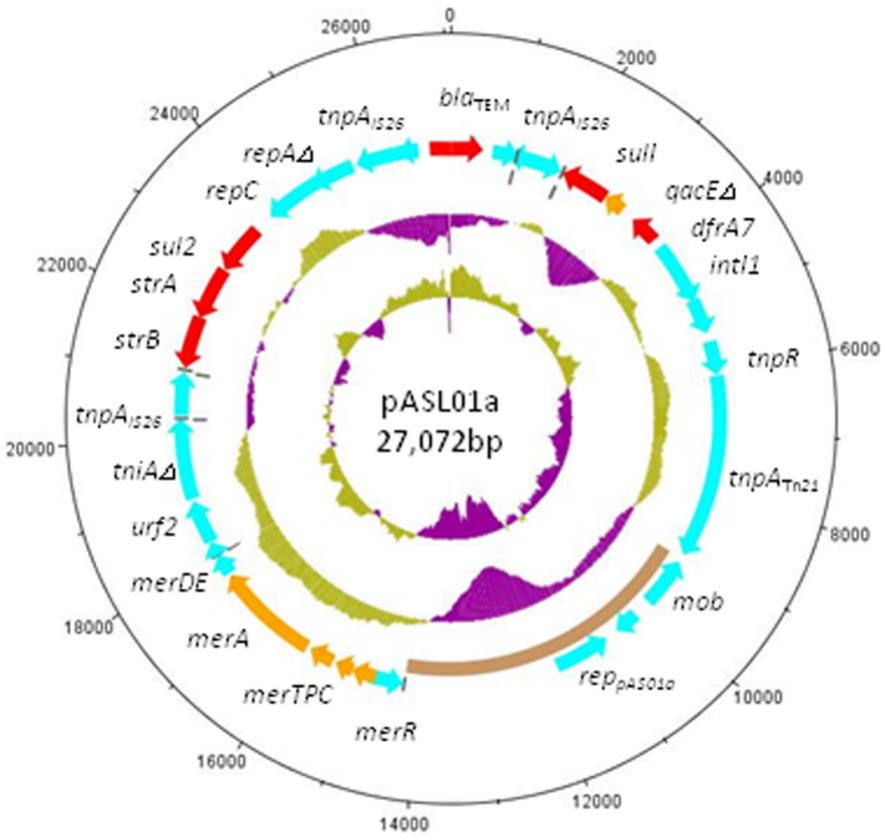
Circular map of 27,072bp pASL01a. Circles display (from the inside) (a) GC skew ([G + C]/[G - C]) in a 1,000-bp window (b) GC content in a 1,000-bp window (purple representing above average and yellow below average in both cases) (c) plasmid backbone in beige (d) predicted coding sequences and (e) kilobase scale. Open reading frames predicted to encode drug resistance genes are colored red and those that encode resistance to other chemical entities are gold. All other predicted open reading frames are marked in blue. Dark gray lines on the open-reading frame and backbone tracks mark the position of inverted repeats.

We sequenced the *dfrA7*-bearing plasmid from isolate 05/01a, and designated it pASL01a. The complete sequence of this plasmid has been deposited in Genbank (Accession number JQ480155). As shown in Figure 4, pASL01a is a 27,072 bp plasmid largely comprised of a Tn*21*-family transposable element. pASL01a contains 29 predicted open reading frames and has an overall G+C content of 56.67%. The relatively low G+C content 5,168 bp plasmid backbone contains only three open reading frames: the replicon, a putative *mob* gene and an *orf* of unknown function. Although a precise origin of replication could not be identified, a sharp change in GC skew just upstream of repA_AS01a_ indicates the location of the likely origin. Analysis using BLAST revealed that the backbone region is most similar to several unpublished or recently published sequences of small ColE-related plasmids. The best matches (98% or greater identical at the DNA level) include the 5,146 bp *E. coli* plasmid pIGJC156 (gb|EU090225.1|), the 7,462 bp *E. coli* plasmid pMG828-4 (gb|DQ995354.1|) and multiple plasmids from recently genome-sequenced pathogens [72], [73]. Plasmid pIGJC156 is 97% identical to the backbone of pASL01a. The only dissimilar region between pIGJC156 and the pASL01a backbone is the replicon. The replicon of plasmid pMG828-4 is also not significantly similar to that of pASL01a and 1 kb of downstream pASL01a sequence is replaced by a putative NADH oxidase gene not present in pASL01a. The replicons of the pathogen plasmids are over 98% identical to that of pASL01a [72], [73]. None of these plasmids carry any virulence genes and all are present in strains that carry other, larger plasmids.

pASL01a is principally comprised of a transposon with the basic transposition module and mercury resistance genes of Tn*21*. With the exception of the In*2*-associated *tni* module, which is truncated in pASL01a, all the transposition components of Tn*21* are present and conserved with the transposon, suggesting that the element may be mobile. Between these modules, as in Tn*21*, a class 1 integron(carrying *dfrA7*, *sulI* and *qacEΔ1* genes) is inserted. Between the In2 and *tni* modules of the Tn*21*-like element is inserted an *IS26*-derived resistance element, bearing widely disseminated genes encoding resistance to beta lactams (*blaTEM*), sulphonamides (*sul2*) and aminoglycosides (*strAB*). The gene content and order of this module is identical to that of plasmid pAKU_1 and pHCM1 from *Salmonella enterica* Paratyphi and Typhi respectively [55], [74]. This 9 Kb *bla/sul/str* module has also been encountered on a number of other enterobacterial multidrug resistance plasmids and our data add to the evidence that suggests that the element is highly promiscuous [74], [75], [76] and, as Holt et al [74] speculate, moves as a block. A separate study from Australia proposes that the *IS26*-flanked *sul/str* portion of this transposon, originally described in plasmid RSF1010, may itself be a separable module [77]. The locations of repeats and alterations in G+C content depicted in Figure 4, suggest that any or all of these possibilities may account for our encountering this resistance transposon on a plasmid of unusual backbone from Nigeria. The transposon described here lacks a Tn*9*-derived portion encoding chloramphenicol resistance often seen in resistance plasmids bearing Tn*21*-derived elements and could share an ancestor with those that have mediated rapid acquisition of multiple resistance genes in *Salmonella* [36], [74], [78]. The *dfrA7*-bearing transposon from pASL01a is also in many ways similar to transposon Tn*5086*, a Tn21-family transposon restriction mapped in 1993 [48]. Tn*5086* possesses a Tn*21* backbone, with *dfrA7* and mercury genes included, but lacks the *bla/sul/str* element.

Close examination of the predicted DfrA protein encoded by pASL01a reveals that the gene, and its flanking sequence, is identical to that of Tn*5086* [48]. Sundström et al [48] demonstrated experimentally that the start codon was a TTG, and that the upstream ATG codons that are annotated as starts for many *dfrA7* and *dfrA17* alleles in the Genbank database were in fact not required for translation and overlapped the ribosomal binding site. As there are some *dfrA7* alleles in the database that have an ATG codon at this locus, we resequenced the *dfrA7* gene alone, obtaining 6X sequence coverage from three independent pASL01a subclones, and confirming the TTG codon. TTG start codons are uncommon in *E. coli* and it is therefore unusual that a resistance gene that begins with this codon has been so widely and successfully disseminated. A variant *dfrA7* allele, this time with polymorphisms at the C-terminal end, has been previously reported from Nigeria [63]. The existence of multiple *dfrA7* alleles in this population is likely linked to the high frequency of the gene. However, conservation of the functional regions strongly suggests that the gene remains under selection, consistent with intensive local trimethoprim use [1].

### pASL01a is Only One of Multiple Vectors for TnASL01a

We used PCR to screen the isolates from Nigeria for *repC*, a gene that lies within the predicted transposon as well as rep_ASL01a_ and the putative *mob* locus on pASL01a, which are part of the plasmid backbone. Using primers Lev5’CS (located immediately upstream of *dfrA7*) and JSMrepCR (within the transposon-borne *repC* gene), we verified by long PCR that the distance between these two genes was 6 Kb for all 22 2005 isolates from Nigeria that carry the *dfrA7 and the repC* genes as well as for the three *dfrA7*-positive isolates from Ghana that were employed in conjugation experiments.

Using primers for *intI1* (TnASL01a class 1 integron gene), *repC* from TnASl01a and the region of pASL01a encompassing the plasmid origin of replication and putative *mob* genes (Table S1), we screened the 2005 and 2009 isolates for different regions of pASL01a. As shown in Table 4, only 12 (18.5%) of 65 *dfrA7*-positive isolates from Nigeria possessed the pASL01a *rep-mob* backbone region together with *repC* and *intI1*. However, 36 (55.4%) strains were positive for *repC* and *intI1* but negative for the pASL01a backbone markers. Similarly, we detected *repC* in 37 (64.9%) *dfrA7*-positive strains from Ghana but only four (7.0%) *dfrA7*-positive strains also had the pASL01a backbone genes. Interestingly, the pASL01a plasmid backbone was significantly less common in *dfrA7*-positive isolates from Ghana, compared to isolates from Nigeria, where it was originally identified (p = 0.038, Yates-corrected Chi-squared test). However the *repC* was identified at statistically similar levels at both sites (p = 0.9).

The data demonstrate that while presence of *dfrA7* is strongly associated with Tn*ASL01a*, there are other class 1 integron-borne genes that are likely to be disseminated by Tn21-like transposons that have the *repC* gene (Table 4). These include *aadA1, dfrA1-aadA1, blaOXA* and *dfrA5*, which have previously been reported in the literature and showed similar strong associations in this study (Table 4) [76], [78], [79]. These findings emphasize that transposon-borne integrons are flexible platforms that can evolve to carry different genes, depending on cassette availability, integrase activation and selective pressure. Such a model could account for the origin of the *dfrA7*-bearing transposon we report here and suggests that other cassettes may be incorporated when new antibacterials are introduced.

**Table 4:** Number and percentage of isolates bearing TnASL01a-associated sequences *intI1* and *repC*, and pASL01a backbone associated sequences *rep_ASL01a_* and *mob*.

Country, Year of isolation	Class 1 integron cassettes	*repC, intI1* (without *rep_ASL01a_*, *mob)*	*repC, intI1*and*rep_ASL01a_*, mob*	Neither *repC* nor*rep_ASL01a_*, *mob*
Nigeria, 2005	*dfrA7*	14 (63.6)	7 (31.8)	1 (4.5)
	Other class 1 integron cassettes	5 (33.3)	2 (13.3)	8 (53.3)
	No class 1 integron	8 (9.8)	0 (0)	73 (89.0)
Nigeria, 2009	*dfrA7*	32 (71.1)	5 (11.1)	8 (17.8)
	Other class 1 integron cassettes	19 (65.5)	5 (17.2)	9 (31.0)
	No class 1 integron	30 (42.9)	1 (1.42)	39 (55.7)
Ghana, 2006	*dfrA7*	15 (78.9)	2 (10.5)	0 (0)
	Other class 1 integron cassettes	3 (18.8)	0 (0)	13 (81.3)
	No class 1 integron	19 (22.6)	0 (0)	65 (77.4)
Ghana, 2007	*dfrA7*	9 (81.8)	1 (9.1)	1 (9.1)
	Other class 1 integron cassettes	5 (41.6)	0 (0)	7 (58.3)
	No class 1 integron	8 (17.0)	0 (0)	38 (80.9)
Ghana, 2008	*dfrA7*	13 (54.2)	1 (4.2)	10 (41.7)
	Other class 1 integron cassettes	13 61.9)	0 (0)	8 (38.1)
	No class 1 integron	11 (25.6)	1 (2.3)	31 (72.1)

Plasmids bearing *dfrA7* genes in the context we describe have been reported from *Salmonella enterica* Typhi and Paratyphi serovars [36], [74]. Although integrons are more commonly sought in *E. coli* reports of class 1-integron-borne *dfrA7* alleles are comparatively less common. In *Salmonella*, *dfrA7*-bearing transposons are typically found on large IncH1 plasmids, which have spread through Asia [55], [74]. However we did not identify IncH plasmids in this study and the plasmid isolated from strain 05/01a was relatively small, carrying only the resistance transposon, and a small 5 Kb core. Resistance genes can gain success by hitchhiking on successful self-transmissible elements or entering epidemic bacterial clones, both mechanisms of which have been the focus of most published studies. Resistance genes, such as the *dfrA7* allele we have mapped in this study, can equally achieve prominence by trans-mobilization, either because they are carried on elements that can be mobilized [80] or because they can transpose between mobile and non-mobile elements [81].

### Conclusions

Our work demonstrates that a small repertoire of widely disseminated mobile cassettes account for most of the trimethoprim resistance among *E. coli* isolates in Nigeria and Ghana. Many *dfrA* alleles associated with integrons, transmissible plasmids, or both, are globally disseminated. For example, detection of *dfrA1*-containing integrons in our study is not surprising and indicative of a globally disseminated resistance pool, from which organisms in West Africa are not isolated [82], [83]. We identified a *dfrA7* allele that is extraordinarily over-represented in Nigeria, Ghana and possibly elsewhere in sub-Saharan Africa. The allele lies within a class 1 integron borne on a potentially mobilizable Tn*21*-like transposon.

It is not clear when the *dfrA7*–bearing transposon entered the *E. coli* commensal population in Nigeria because strains isolated prior to 2005 were unfortunately not archived. *dfrA7* was reported as early as 1995 from South Africa when Adrian et al [59] described it as the most common *dfr* allele. Interestingly, they were unable to transfer the *dfrA7* marker in that study by conjugation and southern hybridization demonstrated that the gene was, in most cases, located on the chromosome. Lamikanra and coworkers [57], [84], suggest that the predominant *dfr* allele in the 1980s and early 1990s was a class 2 integron borne *dfrA1,* which we still detect significantly in both Nigeria and Ghana. *dfrA7* was detected in those earlier studies, but less commonly, and was transferable by conjugation [57], [84]. Thus it is possible that the transposon we describe has been in circulation in parts of Africa for some time. However it is tempting to view its introduction, or selective success, as a recent event because we have seen trimethoprim resistance increase rapidly after 2005.

Minimal data on resistance elements from Africa are available because few molecular studies are performed there. Kingsley et al (2009) report that strains belonging to the recently evolved invasive non-typhoidal *Salmonella* lineage isolated in Malawi and Kenya carry resistance genes on Tn*21*-type transposons very similar to that identified in this study [78]. The resistance genes on those transposons largely account for the treatment failure associated with these infections in recent years. Our study opens questions as to how antimicrobial use patterns, or other risk factors, may have provided enormous selective advantage for multi-drug resistance plasmids. Much of Africa has seen unprecedented selective pressure from trimethoprim and sulphamethoxazole in recent times and Skurnik et al (2009) propose that integron-associated resistance is linked to high levels of selective pressure [85]. Since resistance rates remained stable until 1998, we hypothesize that a recent change in antimicrobial use patterns would most likely account for the spread of pASL01a and consequent upsurge in trimethoprim resistance.

Rapid spread of HIV has been coupled with severe resource constraints for managing infected people in Africa. In 1999, the results of a clinical trial revealed that trimethoprim-sulphamethoxazole prophylaxis decreased morbidity and mortality from opportunistic infections in patients in Abidjan, Ivory Coast [86], [87]. These findings resulted in trimethoprim-sulphamethoxazole prophylaxis policies that have been applied almost continent-wide. Much-cited ‘cost effectiveness’ of long-term trimethoprim-sulphamethoxazole prophylaxis for AIDS patients, who, but for slow roll outs, could be on more effective but more expensive antiretrovirals [4], does not factor in the losses from declining trimethoprim and sulphonamide susceptibility due to resistance. In parallel with prophylactic use, this and other recent studies from Africa have reported trimethoprim-resistance rates in clinical isolates of *E. coli* and related organisms that exceed 70% [88], [89], [90]. Studies that specifically examined carriage of resistant *E. coli* and *Streptococcus pneumoniae* among individuals on trimethoprim-sulphamethoxazole prophylaxis, in comparison with those not receiving these drugs, document a significantly greater level of resistance among patients receiving prophylaxis [88], [90], [91]. Trimethoprim- sulphamethoxazole prophylaxis did not select for trimethoprim and sulphonamide resistance alone, but resistance to multiple antimicrobials. In *E. coli*, this is consistent with a role for multiply-resistant elements, such as pASL01a, and the transposon it carries.

In Finland, the rates of trimethoprim resistance among urinary *E. coli* isolates increased from about 10% to 40% between 1978 and 1984, at a time when trimethoprim was used intensively for empiric treatment of urinary tract infections [45]. This rapid increase in resistance was largely due to epidemic dissemination of mobile elements bearing *dfrA1* genes in a scenario similar to *dfrA7*-mediated resistance in West Africa today. When trimethoprim was withdrawn, resistance rates not only failed to decline, they continued to rise because the resistance gene in question was borne on a transposon that could stably integrate into the chromosomes as well as into plasmids [45]. Ultimately, *dfrA1*-bearing transposons were disseminated globally. Because we found that trimethoprim resistance is linked to resistance to other commonly used antimicrobials (penicillins, sulphonamides and streptomycin), we cannot directly implicate trimethoprim use alone in the selection of the transposon identified in this study. In the same vein, we predict that, as was demonstrated in the Finland case and experimentally tested in Kronberg Country Sweden, withdrawal of trimethoprim-sulphamethoxazole is unlikely to be sufficient to produce a return to susceptibility [16], [45]. Our study reveals that gene context is an important determinant of evolutionary success. Since the structure of the transposon demonstrates flexible incorporation of resistance genes, it is essential to control selective pressure to newer agents to prevent their resistance being incorporated into this or other successful platforms.

## Supporting Information

Figure S1 ***Rsa*** **I-based PCR-RFLP of** ***fliC*** **amplicons from fourteen independent** ***dfrA7*** **-bearing isolates demonstrating eleven unique restriction profiles.** Lanes 1, 9 and 18: I Kb ladder plus (Invitrogen).
(PPTX)

Table S1 **Oligonucleotide primers for PCR.**
(DOC)

Table S2 **Resistance to antibiotics 2005 and 2009.**
(DOC)

Table S3 **Allele profiles of** ***dfrA7*** **bearing strains that were multilocus sequenced typed.**
(DOC)
