# Supplementary figures and images for: Regional Dissemination of a Trimethoprim-Resistance Gene Cassette via a Successful Transposable Element
Source: PLoS One. 2012 May 30;7(5):e38142. doi: 10.1371/journal.pone.0038142 (PMC3364232; doi:10.1371/journal.pone.0038142)

## Slide 1
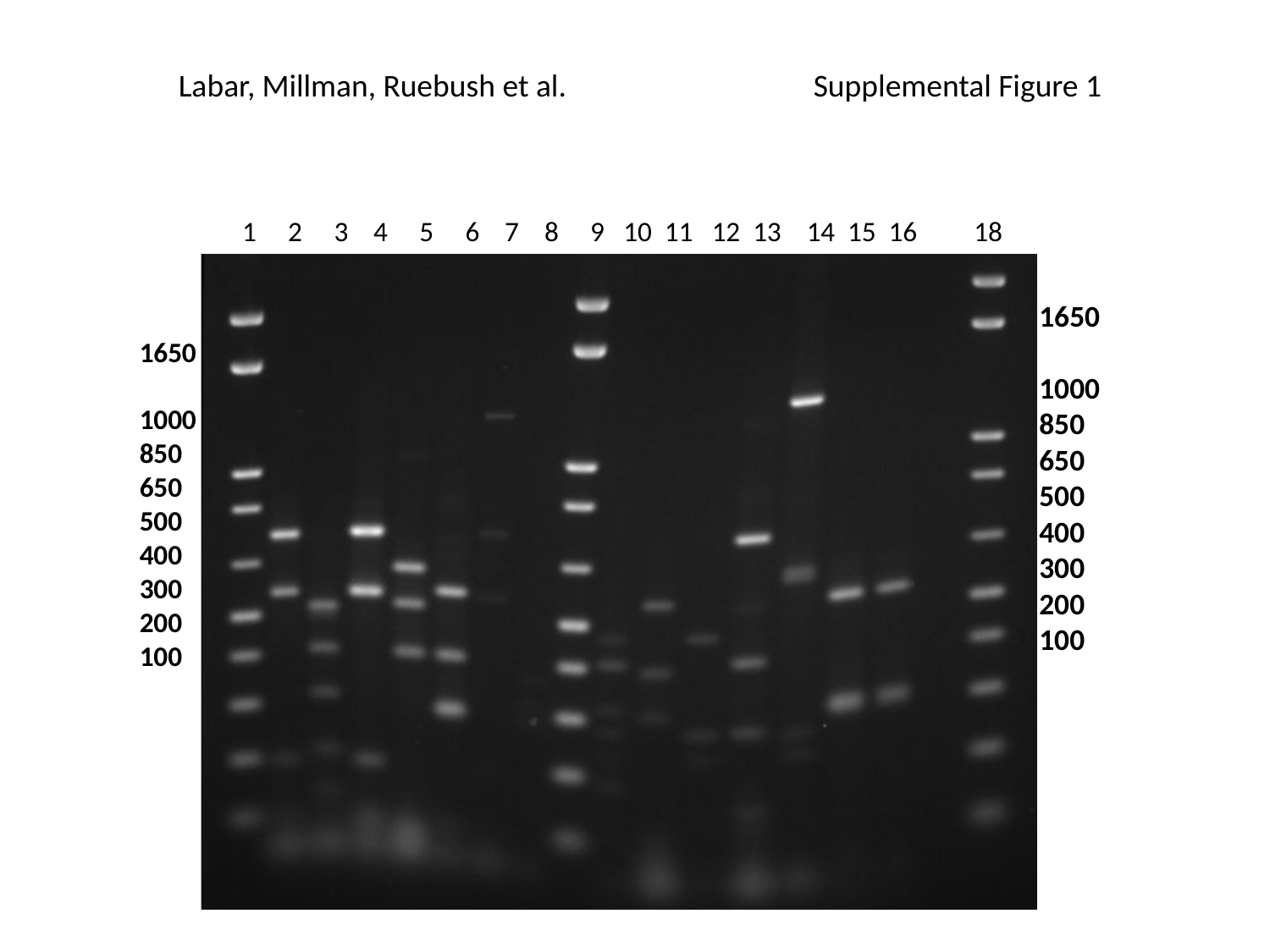

# Labar, Millman, Ruebush et al. 		Supplemental Figure 1
1 2 3 4 5 6 7 8 9 10 11 12 13 14 15 16 18
1650
1000
850
650
500
400
300
200
100
1650
1000
850
650
500
400
300
200
100

Supplement: Figure S1 — Rsa I-based PCR-RFLP of fliC amplicons from fourteen independent dfrA7 -bearing isolates demonstrating eleven unique restriction profiles. Lanes 1, 9 and 18: I Kb ladder plus (Invitrogen). (PPTX) [file pone.0038142.s001.pptx]
