# Supplementary material for: Regional Dissemination of a Trimethoprim-Resistance Gene Cassette via a Successful Transposable Element
Source: PLoS One. 2012 May 30;7(5):e38142. doi: 10.1371/journal.pone.0038142 (PMC3364232; doi:10.1371/journal.pone.0038142)
Supplement: Table S1 — Oligonucleotide primers for PCR. (DOC) [file pone.0038142.s002.doc]

**Table S1:** Oligonucleotide primers used for standard PCR reactions

| **Primer** | **Primer Sequence** | **Purpose, Target locus** | **Amplicon size (bp)** | **Refe-rence** |
| --- | --- | --- | --- | --- |
| Lév5’CS | GGC ATC CAA GCA GCA AG | Class 1 integron, variable region | Variable | (1) |
| Lév3’CS | AAG CAG ACT TGA CCT GA |
| Hep74 | CGG GAT CCC GGA CGG CAT GCA CGA TTT GTA | Class 2 integron, variable region | Variable | (2) |
| Hep51 | GAT GCC ATC GCA AGT ACG AG |
| intI1F | TCT CGG GTA ACA TCA AGG | TnASL01a - Class 1 integron, *intI1* | 500 | (3) |
| intI1R | AGG AGA TCC GAA GAC CTC |
| JSMrepC-F | AGCAAGCTGGACGTGACGTA | TnASL01a, *repC* | 699 | This study |
| JSMrepC-R | CGAACTCGGTTACCGTCCAG |
| p01at035cF | ATCCACGTGACGGTGATTTAA | pASL01a backbone rep_ASL01a_-mob region | 2,555 | This study |
| p01a041cR | CTATCACACGGATGATCTCG |
| F-fliC1 | ATG GCA CAA GTC ATT AAT ACC CAA C | *fliC* internal fragment | Variable | (4) |
| R-fliC2 | CTA ACC CTG CAG CAG AGA CA |
| *adk*F | ATTCTGCTTGGCGCTCCGGG | MLST, *adk* | 583 | (5) |
| *adk*R | CCGTCAACTTTCGCGTATTT |
| *fumC*F | TCACAGGTCGCCAGCGCTTC | MLST,  *fumC* | 806 | (5) |
| *fumC*R | GTACGCAGCGAAAAAGATTC |
| *gyrB*F | TCGGCGACACGGATGACGGC | MLST, *gyrB* | 911 | (5) |
| *gyrB*R | ATCAGGCCTTCACGCGCATC |
| *icd*F | ATGGAAAGTAAAGTAGTTGTTCCGGCACA | MLST, *icd* | 878 | (5) |
| *icd*R | GGACGCAGCAGGATCTGTT |
| *mdh*F | ATGAAAGTCGCAGTCCTCGGCGCTGCTGGCGG | MLST, *mdh* | 932 | (5) |
| *mdh*R | TTAACGAACTCCTGCCCCAGAGCGATATCTTTCTT |
| *purA*F | CGCGCTGATGAAAGAGATGA | MLST, *purA* | 816 | (5) |
| *purA*R | CATACGGTAAGCCACGCAGA |
| *recA*F | CGCATTCGCTTTACCCTGACC | MLST, *recA* | 780 | (5) |
| *recA*R | TCGTCGAAATCTACGGACCGGA |

**References**

1. Lévesque C, Piché L, Larose C, & Roy PH (1995) PCR mapping of integrons reveals several novel combinations of resistance genes. *Antimicrobial Agents and Chemotherapy* 39(1):185-191.

2. White PA, McIver CJ, & Rawlinson WD (2001) Integrons and gene cassettes in the enterobacteriaceae. *Antimicrob Agents Chemother* 45(9):2658-2661.

3. Leverstein-van Hall MA*, et al.* (2003) Multidrug resistance among Enterobacteriaceae is strongly associated with the presence of integrons and is independent of species or isolate origin. *J Infect Dis* 187(2):251-259.

4. Fields P*, et al.* (1997) Molecular characterization of the gene encoding H antigen in *Escherichia coli* and development of a PCR-restriction fragment length polymorphism test for identification of *E. coli* O157:H7 and O157:NM. *Journal of Clinical Microbiology* 35:1066-1070.

5. Wirth T*, et al.* (2006) Sex and virulence in *Escherichia coli:* an evolutionary perspective. *Mol Microbiol* 60(5):1136-1151.
