# Supplementary material for: Regional Dissemination of a Trimethoprim-Resistance Gene Cassette via a Successful Transposable Element
Source: PLoS One. 2012 May 30;7(5):e38142. doi: 10.1371/journal.pone.0038142 (PMC3364232; doi:10.1371/journal.pone.0038142)
Supplement: Table S2 — Resistance to antibiotics 2005 and 2009. (DOC) [file pone.0038142.s003.doc]

**Table S2:** Antimicrobial resistance of *Escherichia coli* strains isolated from healthy individuals in Nigeria in 2005 and 2009

|  | Number (%) resistant | |
| --- | --- | --- |
| Antibacterial | 2005 (n=128) | 2009 (n=172) |
| Trimethoprim | 86 (67.2) | 122 (70.8) |
| Ampicillin | 60 (46.7) | 135 (78.3) |
| Tetracycline | 97 (75.4) | 145 (84.2) |
| Chloramphenicol | 20 (15.6) | 47 (27.5) |
| Nalidixic acid | 20 (15.6) | 36 (20.9) |
| Ciprofloxacin | 1 (0.7) | 11 (6.4) |
| Streptomycin | 116 (91) | 159 (92.4) |
| Sulphonamide | 88 (68.9) | 134 (77.8) |
