# Supplementary material for: Regional Dissemination of a Trimethoprim-Resistance Gene Cassette via a Successful Transposable Element
Source: PLoS One. 2012 May 30;7(5):e38142. doi: 10.1371/journal.pone.0038142 (PMC3364232; doi:10.1371/journal.pone.0038142)
Supplement: Table S3 — Allele profiles of dfrA7 bearing strains that were multilocus sequenced typed. (DOC) [file pone.0038142.s004.doc]

Table S3: Alllele profiles of *dfrA7* bearing strains that were multilocus sequenced typed

| Strain | Country (Year) | adk | fumC | gyrB | icd | mdh | purA | recA | ST | ST complex |
| --- | --- | --- | --- | --- | --- | --- | --- | --- | --- | --- |
| 05/01a | Nigeria (2005) | 10 | 11 | 4 | 8 | 8 | 8 | 2 | 10 | 10 |
| 05/23a | Nigeria (2005) | 10 | 11 | 4 | 8 | 8 | 8 | 2 | 10 | 10 |
| 05/33a | Nigeria (2005) | 10 | 11 | 4 | 8 | 8 | 8 | 2 | 10 | 10 |
| 05/31a | Nigeria (2005) | 106 | 11 | 4 | 8 | 8 | 8 | 2 | 494 |  |
| 05/32c | Nigeria (2005) | 56 | 6 | 5 | 9 | 9 | 8 | 2 | 503 | 13 |
| 05/30a | Nigeria (2005) | 6 | 29 | 32 | 16 | 11 | 8 | 44 | 156 | 156 |
| 05/09c | Nigeria (2005) | 109 | 65 | 5 | 1 | 9 | 13 | 14 | 517 |  |
| 06/079 | Ghana (2006) | 6 | 65 | 2 | 25 | 5 | 5 | 2 | 1466 |  |
| 06/045 | Ghana (2006) | 10 | 174 | 4 | 8 | 8 | 8 | 2 | 1286 | 10 |
| 06/036 | Ghana (2006) | 6 | 8 | 4 | 1 | 9 | 48 | 7 | 210 |  |
| 08/78 | Ghana (2008) | 10 | 11 | 4 | 8 | 8 | 8 | 2 | 10 | 10 |
| 08/91 | Ghana (2008) | 10 | 11 | 4 | 1 | 8 | 9 | 2 | 227 | 1 |
